# Submaximal 2‐day cardiopulmonary exercise testing to assess exercise capacity and post‐exertional symptom exacerbation in people with long COVID

**DOI:** 10.1113/EP092576

**Published:** 2025-06-13

**Authors:** Callum Thomas, Nik Kudiersky, Paul Ansdell, Ruth E Ashton, Calum Brown, Thomas Bewick, Jack Carr, Emily Hume, Padraig Spillane, Elisa Pastorio, Rebecca Owen, Tom Maden‐Wilkinson, Ethan McNeil‐Angopa, Tom Parkington, Ross Arena, Cemal Ozemek, Federico Formenti, Sundar Kumar Veluswamy, Rachita Gururaj, Mark A. Faghy

**Affiliations:** ^1^ Biomedical and Clinical Science Research Theme, School of Human Sciences University of Derby Derby UK; ^2^ Physical Activity, Wellbeing and Public Health Research Group, School of Sport and Physical Activity Sheffield Hallam University Sheffield UK; ^3^ Faculty of Health and Life Sciences Northumbria University Newcastle Newcastle upon Tyne UK; ^4^ Research Centre for Physical Activity, Sport and Exercise Sciences (PASES) Coventry University Coventry UK; ^5^ Department of Respiratory Medicine University Hospitals of Derby and Burton NHS Foundation Trust Derby UK; ^6^ Department of Physical Therapy, College of Applied Health Sciences University of Illinois Chicago Chicago Illinois USA; ^7^ Centre for Human and Applied Physiological Sciences King's College London London UK; ^8^ Nuffield Department of Clinical Neurosciences University of Oxford Oxford UK; ^9^ M. S. Ramaiah College of Physiotherapy M. S. Ramaiah University of Applied Sciences Bengaluru India

**Keywords:** COVID‐19, CPET, long COVID, PESE

## Abstract

Long COVID has a complex pathology and a heterogeneous symptom profile that impacts quality of life and functional status. Post‐exertional symptom exacerbation (PESE) affects one‐third of people living with long COVID, but the physiological basis of impaired physical function remains poorly understood. Sixty‐eight people (age (mean ± SD): 50 ± 11 years, 46 females (68%)) were screened for severity of PESE and completed two submaximal cardiopulmonary exercise tests separated by 24 h. Work rate was stratified relative to functional status and was set at 10, 20 or 30 W, increasing by 5 W/min for a maximum of 12 min. At the first ventilatory threshold (VT1), ${\dot{V}}_{O_{2}}$ was 0.73 ± 0.16 L/min on Day 1 and decreased on Day 2 (0.68 ± 0.16 L/min; *P* = 0.003). Work rate at VT1 was lower on Day 2 (Day 1 vs. Day 2; 28 ± 13 vs. 24 ± 12 W; *P* = 0.004). Oxygen pulse on Day 1 at VT1 was 8.2 ± 2.2 mL/beat and was reduced on Day 2 (7.5 ± 1.8 mL/beat; *P* = 0.002). The partial pressure of end tidal carbon dioxide was reduced on Day 2 (Day 1 vs. Day 2; 38 ± 3.8 vs. 37 ± 3.2 mmHg; *P* = 0.010). Impaired ${\dot{V}}_{O_{2}}$ is indicative of reduced transport and/or utilisation of oxygen. ${\dot{V}}_{O_{2}}$ at VT1 was impaired on Day 2, highlighting worsened function in the 24 h after submaximal exercise. The data suggest multiple contributing physiological mechanisms across different systems and further research is needed to investigate these areas.

## Abstract (highlights)

- **What is the central question of this study?**
  Can a submaximal 2‐day cardiopulmonary exercise test (CPET) protocol suggest why people living with long COVID experience post‐exertional symptom exacerbation (PESE)?
- **What is the main finding and its importance?**
  A submaximal 2‐day CPET protocol revealed a reduction in oxygen uptake, oxygen pulse and partial pressure of end tidal carbon dioxide, suggesting dysfunctional oxygen transport, utilisation or both may contribute to long COVID PESE. Provided that there are appropriate and detailed screening processes that exclude people living with moderate–severe‐risk post‐exertional malaise, submaximal CPET offers a safe and informative option to investigate long COVID pathophysiology.

## 1 INTRODUCTION

Long COVID is defined as persistent symptoms or new symptoms presenting more than 3 months after the onset of an acute COVID‐19 infection (WHO, 2021). Long COVID is a complex pathophysiological condition that affects multiple bodily systems (including but not limited to the respiratory, cardiovascular, nervous and muscular systems) and impairs ‘normal’ function (Al‐Aly et al., 2024). Resulting from this complexity is a broad and heterogeneous profile that is associated with over 200 symptoms (Davis et al., 2021; Greenhalgh et al., 2024), and is highly sensitive and episodic in frequency and severity (Brown & O'Brien, 2021; Callan et al., 2022; Thomas et al., 2023). To date, research has widely demonstrated the impact that long COVID has on quality of life (QoL) and functional status with detailed clinical investigations (Davis et al., 2021; Hanson et al., 2022; Kim et al., 2023; Sarkanen et al., 2023) and lived‐experience accounts (Kennelly et al., 2023; Thomas et al., 2023). With ∼65 million people living with long COVID globally and an absence of established treatments and management pathways, there is a substantive and growing cost to health care services with wide‐ranging social and economic impacts (Cambridge Econometrics, 2024). Whilst the full health, social and economic effects of COVID‐19 and long COVID are not completely understood, the economic burden of chronic illness and declining population health is being realised with COVID‐19/long COVID costing the global economy a conservative £1 trillion per annum (Al‐Aly et al., 2024). Macroeconomic insights into the impacts of long Covid in the UK highlight that 1.6 million people are not able to work because of long COVID, reducing gross domestic product (GDP) by £1.5 billion per annum or 140,000 jobs by 2030 if long COVID cases were to rise to 4 million in the UK (Cambridge Econometrics, 2024).

Consequently, there is an urgent need to establish safe, effective and restorative treatment and management approaches. Some practitioners are recommending physical therapies such as exercise training and rehabilitation to improve patient outcomes (Ladlow et al., 2024). However, to date, there is a paucity of research that demonstrates efficacy of physical therapy across the entire long COVID population, and it is currently not recommended by the World Health Organisation (WHO) and National Institute for Health and Care Excellence (NICE) (Torjesen, 2020; WHO, 2023). Coupled with a lack of evidence, the prevalence of post‐exertional symptom exacerbation (PESE) provides an additional consideration and level of complexity. PESE affects approximately one‐third of people living with long COVID and is defined as a worsening of symptoms that occurs after any exertion above a personal and variable tolerance threshold (Thaweethai et al., 2023). Symptoms of PESE typically start within 48 h of exertion and can last for days and even months, adding further consideration for the development of restorative approaches (Bowe et al., 2023; Faghy, Duncan et al., 2024; Twomey et al., 2022). The mechanisms underpinning PESE and post‐exertional malaise (PEM) in people with long COVID remain unclear, but potential candidates include impaired pulmonary, endothelial, immune, autonomic and mitochondrial function, in addition to dysfunctional blood clotting (Appelman et al., 2024; Davis et al., 2023; Faghy et al., 2020; Faghy, Duncan et al., 2024).

Cardiopulmonary exercise testing (CPET) has an evolving recognition for its importance and diagnostic capabilities within healthcare settings (Paolillo & Agostoni, 2017). Primarily, CPET is used to evaluate the integrative response to incremental exercise (Faghy et al., 2020; Singh et al., 2022), enabling characterisation of cardiorespiratory fitness and reasons for physical impairment (Christle and Arena, 2020). CPET is widely recognised as playing an important role in clinical areas, including many uses such as being able to determine surgical operability, evaluating the risk of perioperative death, post‐operative complications and supporting pre‐operative planning algorithms, and in the context of long COVID, for supporting the development of objective management strategies for complex pathological conditions (Corrà et al., 2018; Faghy et al., 2020; Kallianos et al., 2014; Mezzani, 2017).

To support the mechanistic understanding of long COVID and PESE, 2‐day CPETs may provide greater clinical insight compared to the more traditional single‐test method. Two‐day approaches involve two CPET assessments completed 24 h apart and have the capability to evaluate PESE and similarly related conditions such as long COVID and myalgic encephalomyelitis/chronic fatigue syndrome (ME/CFS). This approach, particularly at submaximal work rates (WRs), enables the objective measurement of people's ability to respond to and tolerate everyday physical stimuli, and then to track recovery and subsequent performance following a secondary physical stimulus. Deleterious effects in the form of severe fatigue have been observed after high‐intensity exercise over several days in people living with long COVID and/or ME/CFS (Loy et al., 2016; Twomey et al., 2022), and a submaximal approach along with appropriate screening for PEM reduces the risk of a severe response, enables better comparisons between days due to variability in ability of clinical populations to reach their maximum, and has greater generalizability to activities of daily life (Cook et al., 2012; Noonan and Dean, 2000; Reed et al., 2020). A meta‐analysis evaluating 2‐day CPETs in people with ME/CFS revealed a reduction in ${\dot{V}}_{O_{2}}$ at the first ventilatory threshold (VT1) and peak ${\dot{V}}_{O_{2}}$ on the second day (Lim et al., 2020). This reduction indicates that a single bout of exercise may impair aerobic metabolism, possibly due to reduced oxygen transport (e.g., lowered cardiac output, dysfunctional haematological factors, and/or skeletal muscle perfusion) and/or oxygen extraction (e.g., mitochondrial dysfunction) in people with ME/CFS. Several studies have demonstrated that exercise capacity is reduced in people with long COVID (using a single exercise test) (Appelman et al., 2024; Durstenfeld et al., 2022). However, only one study has investigated the utility of 2‐day CPET in this population and found just one difference in submaximal CPET parameters between days (a reduction in ${\dot{V}}_{E}/{\dot{V}}_{CO_{2}}$ at the gas exchange threshold on Day 2 compared with Day 1) (Gattoni et al., 2025). Accordingly, this study aimed (1) to assess the safety and utility of two submaximal CPETs 24 h apart, (2) to determine any 24‐h‐long effects of submaximal CPET, and (3) to increase mechanistic understanding of long COVID. It was hypothesised that CPET parameters at submaximal thresholds would be impaired on CPET Day 2 compared with CPET Day 1, and these data could suggest causal mechanisms responsible for PESE. It was also hypothesised that no severe adverse events would be elicited during or after submaximal exercise.

## 2 METHODS

### 2.1 Ethical approval

The study was prospectively registered with clinicaltrials.gov (NCT06394921) and received NHS (IRAS ID: 313936) and institutional (ETH2324‐1808) research ethical approval. Written consent was obtained from all participants, and the study conformed to the standards set by Good Clinical Practice (GCP) and by the *Declaration of Helsinki* (Version 2024).

This was a multi‐centre, cross‐sectional, observational study conducted across three sites in the United Kingdom: the University of Derby, Sheffield Hallam University and Northumbria University. Participants who met the WHO long COVID definition (WHO, 2021) were screened for the severity of PESE and completed three study visits, including a baseline assessment and two submaximal CPET visits separated by 24 h.

### 2.2 Screening and eligibility

Eligibility was assessed via telephone consultation and included adults aged 18 to 77 years old, confirmed previous COVID‐19 infection and confirmed long COVID according to the WHO definition. Sufficient English language comprehension and cognitive ability to understand the study protocol, give informed consent and follow instructions were also required. Exclusion criteria comprised the following: <18 years of age, admitted to or received treatment from intensive care units, unconfirmed COVID‐19 test or no retrospective clinician diagnosis, no confirmed long COVID diagnosis from a healthcare professional, reporting a grade 0 or 1 on the Post‐COVID‐19 Functional Status (PCFS) scale, and reporting a 3 or 4 for symptom frequency and severity on the DePaul symptom screening questionnaire (Cotler et al., 2018).

In accordance with established clinical exercise testing guidelines in conducting CPET (Liguori, 2020), additional exclusion criteria were imposed as part of the study safety screening. These included: (1) unstable angina; (2) uncontrolled hypertension, that is, resting systolic blood pressure (SBP) >180 mmHg, or resting diastolic blood pressure (DBP) >110 mmHg; (3) orthostatic blood pressure (BP) drop of >20 mmHg with symptoms; (4) significant aortic stenosis (aortic valve area 120 bpm); (5) acute pericarditis or myocarditis; (6) decompensated heart failure; (7) third degree (complete) atrioventricular (AV) block without pacemaker; (8) recent (3 months) embolism; (9) acute thrombophlebitis; (10) resting ST segment displacement (>2 mm); (11) uncontrolled diabetes mellitus; (12) severe orthopaedic conditions that would prohibit exercise; (13) severe grade 3 rejection (cardiac transplantation recipients); and (14) other metabolic conditions, such as acute thyroiditis, hypokalaemia, hyperkalaemia or hypovolaemia (unless adequately treated).

Each participant's eligibility was logged and discussed individually with a second reviewer (M.F.) to determine suitability and appropriateness for participation in the study. From this process, eleven individuals were determined to be ineligible for participation for one or more of the following reasons: high risk of PEM (*n* = 9), a severe orthopaedic condition that would prevent cycle exercise (*n* = 1), and a severe cardiac issue (*n* = 1).

### 2.3 Participants

Following confirmation of eligibility, 68 participants (age [mean ± SD]: 50 ± 11 years, 46 females [68%]) signed an informed consent form and were recruited to the study from May 2023 to January 2024; participants’ characteristics are presented in Table 1. People living with long COVID were screened against strict eligibility criteria and recruited following referral or contact with a long COVID clinic in the UK (Derbyshire Community Health Services and Sheffield Teaching Hospitals NHS Foundation Trust) or via research pages of established long COVID social media groups.

**TABLE 1:** Participant characteristics.

Characteristic	Value (*n* = 68)
Age, mean ± SD (years)	50 ± 11
Sex, *n* (%) Male Female	22 (32%) 46 (68%)
Height (cm) Weight (kg) Body mass index, mean ± SD (kg/m^2^)	167 ± 9 80 ± 18 29 ± 7
Vaccinated, *n* (%) Yes No One dose Two doses Three doses >Three doses Unknown	67 (99%) 0 (0%) 2 (3%) 10 (15%) 21 (31%) 34 (51%) 1* (1%)
Occupational status, *n* (%) Employed full time Employed part time Illness absence from work Student Retired	32 (47%) 9 (13%) 15 (22%) 2 (3%) 10 (15%)
Comorbidities, *n* (%) Yes No Endocrine/diabetes Renal Cardiovascular Gastrointestinal/liver Neurological/cerebrovascular Malignancy including haematological Respiratory Rheumatological Psychological Other	48 (71%) 20 (29%) 8 (12%) 3 (4%) 19 (28%) 16 (24%) 21 (31%) 1 (2%) 17 (25%) 7 (10%) 6 (9%) 13 (19%)

### 2.4 Study procedures

#### 2.4.1 Baseline assessment (Visit 1)

**FIGURE 1:**
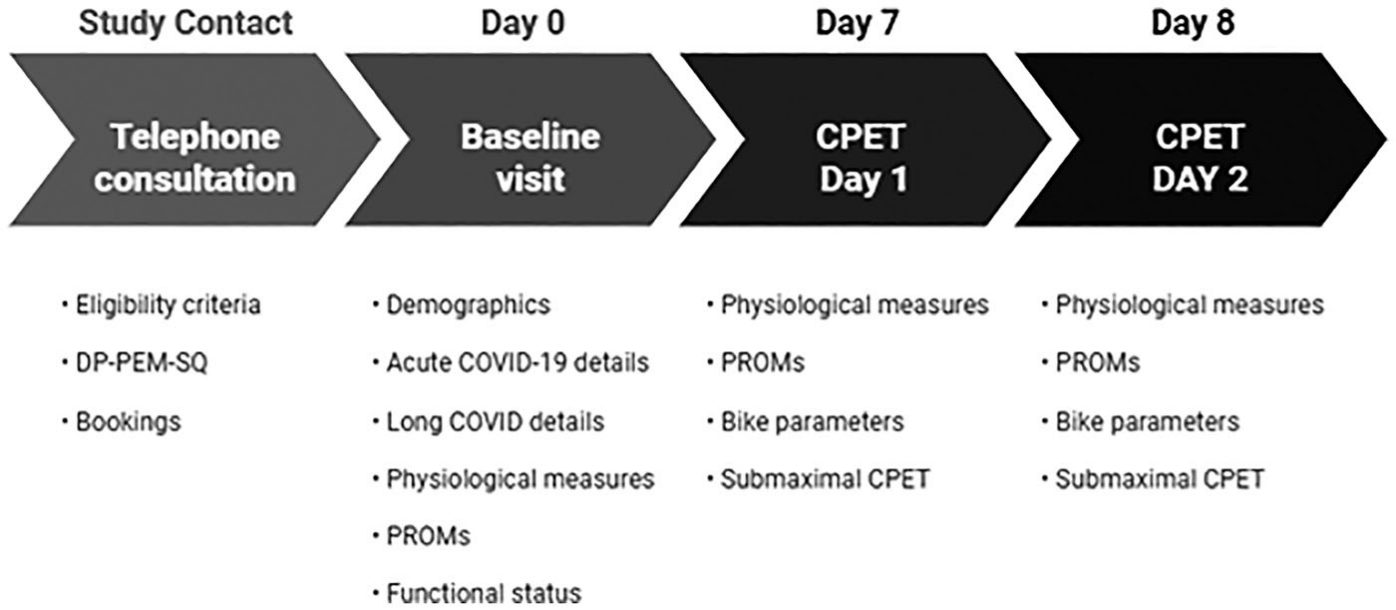
Study timeline.

Following confirmation of eligibility, participants attended three laboratory visits. Baseline data collection (as outlined in Figure 1) consisted of a full demographic profile (age, sex, smoking history and past medical history), detailed acute COVID‐19 history (number of confirmed infections, vaccinations, hospital admissions, acute symptoms, retrospective assessment of performance status, pre‐infection exercise tolerance) and long COVID (diagnosis, symptoms, current access to treatments/services). Physiological observations including heart rate (HR), BP, peripheral oxygen saturation ($S_{pO_{2}}$), respiratory rate, lung function (spirometry), respiratory muscle function (maximal inspiratory (MIP) and maximal expiratory pressure (MEP) and temperature were also captured.

#### 2.4.2 Symptom app reporting

A mobile device app developed by Sheffield Hallam University was used daily to report symptom severity and overall health a week prior to CPET Day 1, and for a week following CPET Day 2. Participants were asked to rate their overall health on a 0–100 scale (100 = best health, 0 = worst health), and the severity of several commonly associated long COVID symptoms such as fatigue, breathlessness, and difficulty thinking on a 0–100 scale (100 = high severity, 0 = symptom not present).

#### 2.4.3 Patient‐reported outcome measures

Patient‐reported outcome measures (PROMs) were collected at all three study visits (described below). These were administered via paper form and delivered by researchers in the baseline assessment. For subsequent visits and to manage cognitive and emotional load, people living with long COVID were provided with physical copies of each PROM to take home and requested to complete these on the morning of each follow‐up visit. All PROMs were checked for completeness at the start of subsequent visits, and participants were allowed to seek clarification, ask questions and complete any outstanding questions.

#### 2.4.4 PCFS scale

The PCFS scale evaluates the ultimate consequences of COVID‐19 on functional status and supplements other instruments that measure QoL, tiredness or dyspnoea in the acute phase. The PCFS covers the full spectrum of functional outcomes and focuses on both limitations in usual duties/activities and changes in lifestyle in six scale grades (Klok et al., 2020). Symptom profile was measured twice at the baseline assessment to capture: (1) retrospective assessment by the patient of their symptom status at symptom onset (essentially their baseline symptoms); and (2) their current symptom status at the point of testing. The impact of current symptom status on daily life on a scale of 0–10 was also measured.

#### 2.4.5 Symptom score

The Symptom Score, an 18‐item scale, asked people living with long COVID to report symptoms that have bothered them within the last 24 h on a scale of 0–5 (0, no symptoms; 5, extremely bothered).

#### 2.4.6 EuroQol 5‐Dimension 5‐Level

QoL was assessed using the EuroQol 5‐Dimension 5‐Level (EQ‐5D‐5L) which is routinely used in the assessment of the QoL in respiratory research and includes a visual analogue score (VAS) of 0–100, 0 being the worst health you can imagine and 100 being the best health you can imagine (Herdman et al., 2011).

#### 2.4.7 Fatigue Assessment Scale and Modified Fatigue Impact Scale

Fatigue is a common complaint in people with long COVID (Davis et al., 2021; Faghy et al., 2022; Owen et al., 2023; Thomas et al., 2023) and is not adequately captured in general QoL or specific recovery questionnaires. Accordingly, participants completed two separate measures of fatigue: (1) the Fatigue Assessment Scale (FAS), a self‐reported questionnaire validated to assess the presence and severity of fatigue (Michielsen et al., 2003); and (2) the Modified Fatigue Impact Scale (MFIS), a 21‐item self‐reported questionnaire assessing fatigue across the physical, cognitive and psychosocial domains (Larson, 2013).

#### 2.4.8 Medical Research Council Dyspnoea Scale

The Medical Research Council (MRC) Dyspnoea Scale was administered to grade the effect of breathlessness on the person's daily activities (Bestall et al., 1999).

#### 2.4.9 Assessment of functional status

Functional status was assessed via the 6‐min walk test (6MWT) and was conducted according to American Thoracic Society guidelines (Holland et al., 2014). The 6MWT is a standardised and widely used measure of functional status in individuals with chronic diseases such as chronic obstructive pulmonary disease, cystic fibrosis, congestive heart failure, peripheral vascular disease and advanced age (Casanova et al., 2011; Ubuane et al., 2018). Data from the 6WMT was used to determine the starting work rate (WR) for an adapted CPET test (strata outline below). In accordance with pilot data (Gururaj et al., 2024), the starting WR was based on 6MWT distance, as follows:

- Stratum I: 6MWT distance <350 m (starting WR of 10 W; with subsequent increments of 5 W).
- Stratum II: 6MWT distance 350–400 m (starting WR of 20 W with subsequent increments of 5 W).
- Stratum III: 6MWT distance >400 m (starting WR of 30 W with subsequent increments of 5 W).

##### 2.4.9.1 CPET tests (Visits 2 and 3)

CPETs completed on Visits 2 and 3 were identical and used an incremental exercise ramp test in accordance with the American Thoracic Society guidelines (Christle and Arena, 2020). Participants completed a submaximal exercise test on a friction‐loaded cycle ergometer (Monark 894E Ergomedic Peak Bike, Monark, Varberg, Sweden), which was calibrated according to manufacturer recommendations before the study. The cycle ergometer was set up according to each participant's body size and personal preference. Seat height and handlebar positions were recorded on CPET Day 1 and replicated on CPET Day 2.

The exercise protocol began with a 3‐min rest period, followed by a maximum of 12 min of exercise delivered via a stepwise incremental protocol. The exercise protocol was individualised based on participants’ predicted exercise capacity, as described above. At the end of the exercise, test participants completed unloaded pedalling and seated rest. Test termination criteria included volition of the participant if symptoms were becoming exacerbated beyond known personal safe limits and cadence dropping below 60 revolutions per minute despite encouragement. Expired respiratory gases, ventilatory profile and electrocardiogram (12‐lead ECG) were analysed continuously. BP was measured at baseline and 2‐min intervals during exercise and recovery. Rating of perceived exertion (RPE; 6 to 20 scale) was measured (Borg, 1998) during the final 15‐s period of each minute. Blood lactate was measured (Lactate Plus, Nova Biomedical, Runcorn, UK) via capillary sampling methods and was completed at the start and end of the test. All data were exported for offline analysis using Microsoft Excel.

### 2.5 Patient and public involvement and engagement statement

Patient and public involvement and engagement (PPIE) was a crucial part of the research design and data collection reported in this manuscript. Previous research team experiences understanding the lived experience of people living with long COVID and CPET testing with chronic disease populations informed the data collection materials and design of this study. A team of PPIE representatives were involved in co‐creating the lab space that mitigated risks of further COVID‐19 infections, raising awareness of our research to recruit participants in their long COVID networks, and PPIE members will also be involved in the dissemination of the results by sharing the findings with their support groups and networks.

### 2.6 Data processing and statistical analysis

CPET and questionnaire data were reviewed for completeness by respective test site coordinators (C.T., N.K., E.H.) (Supporting information). Raw gas analysis data were transformed from breath‐by‐breath to 30 s moving average and middle 5‐of‐7 breath averages to reduce variability and identify data outliers or missing data. Middle 5‐of‐7 breaths data were used to determine the ventilatory threshold (i.e., VT1) and respiratory compensation point (i.e., VT2) using the V‐slope method (Liguori, 2020). Briefly, to determine VT1, carbon dioxide production (${\dot{V}}_{CO_{2}}$) data were plotted as a function of ${\dot{V}}_{O_{2}}$ data in Microsoft Excel. The inflection whereby ${\dot{V}}_{CO_{2}}$ increased disproportionately relative to ${\dot{V}}_{O_{2}}$ indicated VT1. To determine VT2, minute ventilation (${\dot{V}}_{E}$) data were plotted as a function of ${\dot{V}}_{CO_{2}}$ data. The inflection whereby ${\dot{V}}_{E}$ increased disproportionately relative to ${\dot{V}}_{CO_{2}}$ indicated VT2 (Kinnear and Hull, 2021). The ventilatory equivalent method was used to confirm threshold measurements. Ventilatory equivalents represent the ratio of ventilation to oxygen consumption (${\dot{V}}_{E}/{\dot{V}}_{O_{2}}$) and carbon dioxide production (${\dot{V}}_{E}/{\dot{V}}_{CO_{2}}$). VT1 occurs at the nadir of ${\dot{V}}_{E}/{\dot{V}}_{O_{2}}$, whereas VT2 occurs at the onset of a sharp rise in ${\dot{V}}_{E}/{\dot{V}}_{CO_{2}}$. Exercise threshold decisions were checked between test site coordinators with a minimum of 10% compared between sites. The chief investigator (M.F.) resolved any uncertainty on exercise threshold decisions through discussion and agreed collectively where consensus could not be met between site coordinators. The 30 s average data were used for baseline, peak and iso‐time peak calculations whereby iso‐time peak was defined as WRs that were matched within participants for total duration of cycle exercise (comparison of the shortest duration reached of the two datasets) (Curtis et al., 2015; Nicolò et al., 2021).

Normal distribution checks were assessed with skewness and kurtosis scores, as well as *Z*‐scores and inspection of histograms. Data are presented as means ± standard deviation. Parametric data were assessed using a paired samples Student's *t*‐test; non‐parametric data were assessed using Wilcoxon's signed rank test and Freidman's test in SPSS (Version 29, IBM Corp., Armonk, NY, USA) with a set α‐level of 0.050 (two‐tailed; Bonferroni corrected 0.013). Uncorrected *P*‐values are presented within text and tables, and the Bonferroni corrected α‐level for significance was applied for all comparisons. Cohen's *d* was used to calculate the effect size of parametric paired samples *t*‐tests with thresholds set at 0.2 = small, 0.5 = medium and 0.8 = large. To calculate the effect size value of Wilcoxon's signed‐rank *t*‐tests, the formula *Difference between sums of ranks/Total of sums of ranks* was implemented with thresholds set at 0.1 = small, 0.3 = medium and 0.5 = large. To achieve a medium effect with an α‐level of 0.05, with an effect size of 0.35, a minimum of 64 participants were required to demonstrate changes in QoL (measured via EQ‐5D‐5L). A total of 68 participants were included as part of the statistical analysis; meanwhile, it was possible to identify and include 39 participants across both CPET days for analysis at VT1. In accordance with the literature, multiple imputation (MI) was designated for cases where 5–10% of data were missing (Lee and Huber, 2021). However, we did not use it in cases where VT1 and VT2 decisions could not be obtained due to the participant not having reached or exceeded the threshold.

## 3 RESULTS

### 3.1 Participants

#### 3.1.1 Acute COVID

The number of SARS‐CoV‐2 infections (mean ± SD) recorded was 2 ± 1 (range: 1–5 infections from 2020–2023). At least 52% of the cohort had experienced two or more SARS‐CoV‐2 infections; all participants had been vaccinated, with 82% (*n* = 55) receiving at least three doses. One or more comorbidities were experienced by 71% of participants, and 47% of participants were still in full‐time employment (Table 1).

#### 3.1.2 Long COVID

##### PROMs

**FIGURE 2:**
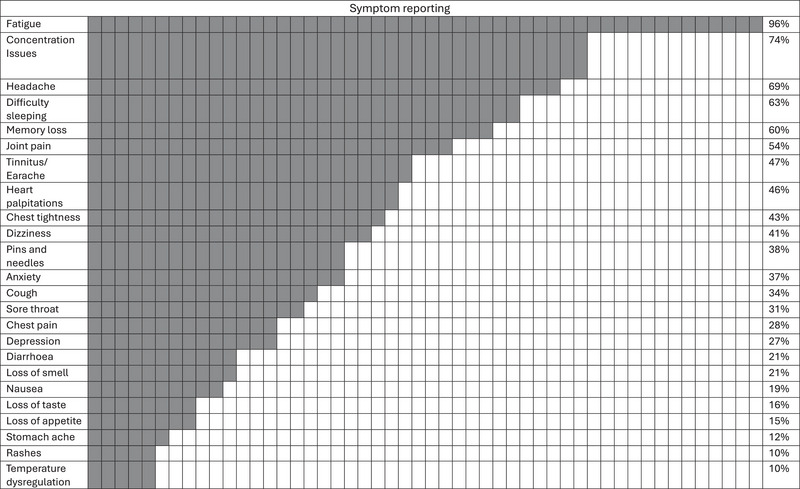
Symptom reporting heatmap showing percentage frequency of symptoms reported by participants at the baseline visit.

The most prevalent symptoms reported at baseline were fatigue (96%), concentration issues (74%) and headaches (69%); however, symptom profile varied across the cohort with the involvement of other physiological systems presenting through difficulty sleeping (63%), joint pain (54%) and heart palpitations (46%) (Figure 2). The impact of symptoms on daily life was rated moderate [*n* = 68] (6 ± 2 out of 10). Overall health observed through the EQ‐5D‐5L utility score and VAS scale at baseline was 0.73 ± 0.16 AU and 55 ± 17 AU, respectively. Baseline status is described relative to various PROMs; PCFS was 3 ± 1 AU, symptom score was 23 ± 10 AU, FAS score was 31 ± 7 AU, MFIS total score was 54 ± 14 AU, MRC dyspnoea scale value was 2 ± 1 AU and cognitive function was 27 ± 2 AU.

##### Symptom app data

Nineteen participants provided responses via the mobile symptom app. Forty‐nine participants did not use the symptom app due to technical issues and non‐compliance with reporting symptoms. No adverse responses were reported or identified during the 7‐day symptom‐reporting and there were no differences in individual symptom severity between baseline and 7 days post‐CPET Day 2. Overall health had decreased 7 days post‐CPET Day 2 compared with baseline; however, this was non‐significant when Bonferroni corrected (Day 1 vs. Day 2 [*n* = 19]; 46 ± 11% vs. 41 ± 16%; *P* = 0.027).

##### Functional status

Resting mean SBP and DBP at baseline for participants were 129 ± 18 and 86 ± 11 mmHg, respectively. Resting HR was 73 ± 13 bpm, and $S_{pO_{2}}$ was 98 ± 1%. Forced expiratory volume in 1 s (FEV_1_) was 2.93 ± 0.78 L, forced vital capacity (FVC) was 3.65 ± 0.97 L, FEV_1_/FVC was 82 ± 6% and peak expiratory flow (PEF) was 452 ± 118 L/min. MIP and MEP pressure were 92 ± 34 cmH_2_O and 120 ± 45 cmH_2_O (means ± SD; *n* = 66), respectively. Mean distance covered by participants on the 6MWT was 418 ± 103 m (range: 206–670 m). Accordingly, 17 (25%), 14 (21%) and 37 (54%) participants were assigned to stratum 1, 2, and 3 of the CPET protocol, respectively.

##### Safety and tolerability

One severe adverse event was reported following completion of Day 1 CPET, where the patient reported a serious exacerbation of symptoms on the day following exercise. Following an investigation, additional steps and mitigation strategies were put in place by the study team to prevent further occurrences; for example, the consequences with regard to health and possible hospitalisation at the extreme, by providing inaccurate information/answers, were emphasised during the study contact. Nine participants (13%) were unable to finish a complete 12‐min submaximal CPET, and five participants (7%) finished only one complete 12‐min submaximal CPET. Reasons for non‐completion on 14 separate CPET sessions were all driven by symptom exacerbation, which included increased breathlessness, fatigue, dizziness and heaviness in the legs. Of this total, two participants reported a history of respiratory complications, which included chronic obstructive pulmonary disease and a history of pneumonia, and non‐completion in these participants was driven by breathlessness. Six participants who did not complete a CPET on at least one day reported a history of cardiovascular complications, which included mild mitral valve prolapse, heart palpitations and ectopic beats, and non‐completion in these participants was driven by heaviness in legs, dizziness and breathlessness. None of the participants met the typical criteria to indicate they had reached their maximum (i.e., a plateau in the ${\dot{V}}_{O_{2}}$ data, blood lactate exceeding 8 mmol/L and a respiratory exchange ratio above 1.0) (Edvardsen et al., 2013), and as such none of the tests were classified as maximal and instead were symptom limited.

##### Rest

Except for the partial pressure of end tidal carbon dioxide ($P_{\mathrm{ETC}O_{2}}$), which was reduced on Day 2 when compared with Day 1 (Day 1 vs. Day 2 [*n* = 68]; 33 ± 3.4 vs. 32 ± 3.4 mmHg; *P* = 0.007), there were no between‐day differences for any of the variables at rest (*P *> 0.050) (Table 2).

**TABLE 2:** Cardiopulmonary resting measures for CPET Days 1 and 2.

	Day 1	Day 2	*P*	Effect size
${\dot{V}}_{O_{2}}$ (L/min)	0.31 ± 0.07	0.32 ± 0.07	0.662	−0.062
${\dot{V}}_{O_{2}}$ (mL/kg/min)	3.96 ± 0.72	4.03 ± 0.86	0.425	−0.111
${\dot{V}}_{O_{2}}$_HR (mL/beat)	4.40 ± 1.05	4.39 ± 1.09	0.418	0.113
HR (bpm)	73 ± 13	74 ± 14	0.038^a^	−0.296
${\dot{V}}_{E}/{\dot{V}}_{O_{2}}$	26.7 ± 5.5	26.8 ± 4.8	0.673	−0.059
${\dot{V}}_{E}/{\dot{V}}_{CO_{2}}$	30.8 ± 5.2	31.0 ± 5.1	0.537	−0.086
RER	0.86 ± 0.08	0.87 ± 0.06	0.414	−0.115
${\dot{V}}_{E}$ (L/min)	10.3 ± 2.5	10.5 ± 2.3	0.257	−0.158
BF (breaths/min)	15 ± 4	16 ± 4	0.025^a^	−0.315
${\dot{V}}_{CO_{2}}$ (L/min)	0.27 ± 0.06	0.27 ± 0.06	0.413	−0.114
$P_{\mathrm{ET}O_{2}}$ (mmHg)	110 ± 7	112 ± 7	0.070	−0.256
$P_{\mathrm{ETC}O_{2}}$ (mmHg)	33 ± 3.4	32 ± 3.4	0.007^b^	0.335
Blood lactate (mmol/L) (*n* = 64)	1.3 ± 0.5	1.4 ± 0.6	0.370	−0.133

*Note*: Values are means ± standard deviation; *n* = 68 LC patients. ^a^Significance at 0.05 α‐level. ^b^Significance with Bonferroni correction (α‐level 0.013). BF, breathing frequency; HR, heart rate; $P_{\mathrm{ET}O_{2}}$ and $P_{\mathrm{ETC}O_{2}}$, end‐tidal oxygen and carbon dioxide, respectively; RER, respiratory exchange ratio; ${\dot{V}}_{CO_{2}}$, carbon dioxide production; ${\dot{V}}_{E}$, minute ventilation; ${\dot{V}}_{E}/{\dot{V}}_{O_{2}}$, the ventilatory equivalent for oxygen; ${\dot{V}}_{E}/{\dot{V}}_{CO_{2}}$, the ventilatory equivalent for carbon dioxide; ${\dot{V}}_{O_{2}}$, oxygen uptake; ${\dot{V}}_{O_{2}}$_HR, oxygen pulse.

#### 3.1.3 Summary of performance/end‐test measures

Table 3 shows the results recorded at iso‐time peak performance. There were no between‐day differences in any of the CPET variables studied at iso‐time peak or end‐test measures (*P *> 0.05). Peak iso‐time WR across both days was 74 ± 14 W. Peak blood lactate was not significantly different between days (Day 1 vs. Day 2 [*n* = 65]; 3.3 ± 1.6 vs. 3.2 ± 1.6 mmol/L; *P* = 0.349).

**TABLE 3:** Cardiopulmonary measures at iso‐time peak for CPET Days 1 and 2.

	Day 1	Day 2	*P*	Effect size
${\dot{V}}_{O_{2}}$ (L/min)	1.31 ± 0.23	1.30 ± 0.21	0.313	0.143
${\dot{V}}_{O_{2}}$ (mL/kg/min)	16.8 ± 3.8	16.8 ± 4.0	0.310	0.142
${\dot{V}}_{O_{2}}$_HR (mL/beat) (*n* = 67)	10.8 ± 2.6	10.7 ± 2.8	0.121	0.218
HR (bpm)	124 ± 24	126 ± 24	0.626	−0.071
${\dot{V}}_{E}/{\dot{V}}_{O_{2}}$ (AU)	30.4 ± 5.4	30.2 ± 5.1	0.157	0.197
${\dot{V}}_{E}/{\dot{V}}_{CO_{2}}$ (AU)	30.6 ± 4.7	30.5 ± 4.4	0.231	0.167
RER	0.99 ± 0.06	0.99 ± 0.06	0.272	0.155
${\dot{V}}_{E}$ (L/min)	43.3 ± 11.7	42.6 ± 10.2	0.250	0.162
BF (breaths/min)	26 ± 8	26 ± 9	0.798	0.036
${\dot{V}}_{CO_{2}}$ (L/min)	1.30 ± 0.25	1.28 ± 0.23	0.224	0.172
$P_{\mathrm{ET}O_{2}}$ (mmHg)	111 ± 9	109 ± 8	0.462	0.103
$P_{\mathrm{ETC}O_{2}}$ (mmHg)	37 ± 4.9	37 ± 4.2	0.758	0.037

*Note*: Values are means ± standard deviation; *n* = 68 LC patients. BF, breathing frequency; HR, heart rate; $P_{\mathrm{ET}O_{2}}$ and $P_{\mathrm{ETC}O_{2}}$, end‐tidal oxygen and carbon dioxide, respectively; RER, respiratory exchange ratio; ${\dot{V}}_{CO_{2}}$, carbon dioxide production; ${\dot{V}}_{E}$, minute ventilation; ${\dot{V}}_{E}/{\dot{V}}_{CO_{2}}$, the ventilatory equivalent for carbon dioxide; ${\dot{V}}_{E}/{\dot{V}}_{O_{2}}$, the ventilatory equivalent for oxygen; ${\dot{V}}_{O_{2}}$, oxygen uptake; ${\dot{V}}_{O_{2}}$_HR, oxygen pulse.

### 3.2 Reduced exercise capacity 24 h after submaximal exercise

#### 3.2.1 Ventilatory thresholds

Cardiopulmonary measures at VT1 for CPET on Days 1 and 2 are presented in Table 4. VT1 was identifiable on both CPET days for 39 participants. Compared with Day 1, at VT1, on Day 2 ${\dot{V}}_{O_{2}}$ was 7% lower (0.68 ± 0.16 L/min; *P* = 0.003), WR was 16% lower (24 ± 12 W; *P* = 0.004), oxygen pulse was 9% lower (7.5 ± 1.8 mL/beat; *P* = 0.002) and $P_{\mathrm{ETC}O_{2}}$ was 3% lower (37 ± 3.2 mmHg; *P* = 0.010).

**TABLE 4:** Cardiopulmonary measures at the first ventilatory threshold for CPET Days 1 and 2.

	Day 1	Day 2	*P*	Effect size
${\dot{V}}_{O_{2}}$ (L/min)	0.73 ± 0.16	0.68 ± 0.16	0.003^b^	0.544
${\dot{V}}_{O_{2}}$ (mL/kg/min)	9.6 ± 2.2	9.1 ± 2.3	0.008^b^	0.490
${\dot{V}}_{O_{2}}$_HR (mL/beat)	8.2 ± 2.2	7.5 ± 1.8	0.002^b^	0.566
HR (bpm)	92 ± 16	92 ± 15	0.531	0.118
Work rate (W)	28 ± 13	24 ± 12	0.004^b^	0.742
${\dot{V}}_{E}/{\dot{V}}_{O_{2}}$ (AU)	22.8 ± 2.4	22.8 ± 2.8	0.925	−0.018
${\dot{V}}_{E}/{\dot{V}}_{CO_{2}}$ (AU)	28.7 ± 3.2	28.7 ± 3.3	0.948	−0.012
RER	0.80 ± 0.05	0.80 ± 0.05	0.786	0.051
${\dot{V}}_{E}$ (L/min)	19.0 ± 3.9	18.1 ± 4.2	0.020^a^	0.435
BF (breaths/min)	19 ± 5	20 ± 6	0.247	−0.213
${\dot{V}}_{CO_{2}}$ (L/min)	0.58 ± 0.14	0.54 ± 0.14	0.033^a^	0.392
$P_{\mathrm{ET}O_{2}}$ (mmHg)	102 ± 7	103 ± 6	0.300	−0.190
$P_{\mathrm{ETC}O_{2}}$ (mmHg)	38 ± 3.8	37 ± 3.3	0.010^b^	0.436

*Note*: Values are means ± standard deviation; *n* = 39 LC patients. ^a^Significant at the 0.05 α‐level only. ^b^Significance with Bonferroni correction (α‐level 0.013). BF, breathing frequency; HR, heart rate; $P_{\mathrm{ET}O_{2}}$ and $P_{\mathrm{ETC}O_{2}}$, end‐tidal oxygen and carbon dioxide, respectively; RER, respiratory exchange ratio; ${\dot{V}}_{CO_{2}}$, carbon dioxide production; ${\dot{V}}_{E}$, minute ventilation; ${\dot{V}}_{E}/{\dot{V}}_{CO_{2}}$, the ventilatory equivalent for carbon dioxide; ${\dot{V}}_{E}/{\dot{V}}_{O_{2}}$, the ventilatory equivalent for oxygen; ${\dot{V}}_{O_{2}}$, oxygen uptake; ${\dot{V}}_{O_{2}}$_HR, oxygen pulse.

**FIGURE 3:**
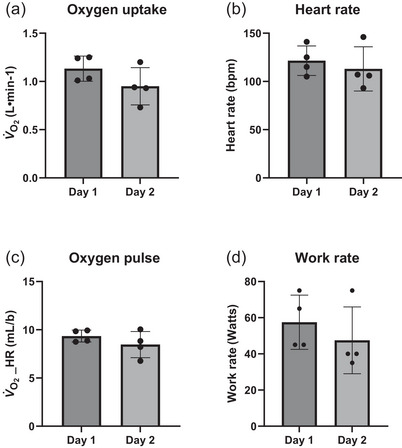
CPET parameters at the respiratory compensation point. (a) Oxygen uptake at VT2 on CPET Day 1 compared with CPET Day 2. (b) HR at VT2 on CPET Day 1 compared with CPET Day 2. (c) Oxygen pulse at VT2 on CPET Day 1 compared with CPET Day 2. (d) Work rate at VT2 on CPET Day 1 compared with CPET Day 2. HR, heart rate.

Cardiopulmonary measures at VT2 for CPET on Days 1 and 2 are presented in Figure 3. VT2 was identifiable on both CPET days for four participants. ${\dot{V}}_{O_{2}}$ at VT2 on Day 1 was 1.13 ± 0.13 L/min and was lower on Day 2 (0.95 ± 0.19 L/min). HR at VT2 was 122 ± 15 bpm on Day 1 and was reduced on Day 2 (113 ± 23 bpm). WR at VT2 on Day 1 reached 58 ± 15 W, and this was lower on Day 2 (48 ± 18 W). Oxygen pulse on Day 1 at VT2 was 9.4 ± 0.6 mL/beat, and this was reduced on Day 2 (8.5 ± 1.4 mL/beat). Respiratory exchange ratio was 1.00 ± 0.02 on Day 1 and was similar on Day 2 (0.98 ± 0.03) at VT2.

## 4 DISCUSSION

This study has demonstrated that people with long COVID without moderate–severe risk of PESE, as determined by the DePaul symptom screening questionnaire, demonstrated an impaired physiological response to submaximal exercise. The key contributing factors to exercise impairment on Day 2 were a reduction in oxygen uptake at submaximal thresholds, accompanied by reduced oxygen pulse and $P_{\mathrm{ETC}O_{2}}$, suggesting dysfunctional oxygen transport, utilisation, or both. As a result, this cohort demonstrated an earlier onset of use of non‐oxidative energy pathways to submaximal exercise. These data highlight physiological mechanisms that could underpin symptoms associated with PEM/PESE in long COVID.

In addition, this study demonstrates the feasibility and safety of adopting a submaximal CPET design across 2 days separated by 24 h. This has helped explore the ability of people living with long COVID to respond and tolerate physical stimuli, track cardiorespiratory parameters to better understand recovery and subsequent performance following a secondary physical stimulus, and gather more information about potential mechanisms for PESE/PEM. Although it is very likely that the deficits in performance and function would be of a greater magnitude if exercise was completed to volitional exhaustion, the data here provide sufficient evidence of multi‐system physiological impairment, whilst maintaining patient safety and wellbeing. This is an important finding as the debilitating effects of PESE/PEM can last for days, weeks and in the worst cases, months (Twomey et al., 2022). These findings can be used to inform pathophysiological understanding, pharmacological and non‐pharmacological intervention strategies, and future research in this area.

The findings from this study indicate that there is an increased dependency on non‐oxidative energy sources during low‐intensity exercise for people living with long COVID early into the protocol, and in many cases, as early as the first minute. In a healthy state, submaximal exercise should primarily be fuelled by aerobic metabolism (Faghy, Tatler et al., 2024). However, of the available literature, which has predominantly looked at maximal testing, CPET research involving people living with long COVID has reported reductions in peak oxygen consumption (Barbagelata et al., 2022; Barker‐Davies et al., 2023; Durstenfeld et al., 2022; Singh et al., 2022; Sørensen et al., 2024). Meanwhile, both maximal and submaximal testing approaches have revealed a lower oxygen consumption at the ventilatory threshold, indicating an increased reliance on non‐oxidative energy provision. For example, Barbagelata et al. (2022) report a lower peak ${\dot{V}}_{O_{2}}$ during incremental treadmill exercise and a higher probability of presenting symptoms during the CPET. However, no previous study has both evaluated a submaximal protocol and performed an additional CPET 24 h later.

A recent publication provides a useful and comprehensive illustration of the compartments of the cardiorespiratory system involved in performing physical activities, from an aerobic and anaerobic perspective (Arena et al., 2022). This model has been reproduced to show where dysfunction may reside in people living with long COVID; specifically, the integrity of the cardiopulmonary systems and the transport and delivery of oxygen in maintaining bodily functions (Faghy, Duncan et al., 2024). This could help identify where pathophysiology resides and what is responsible for the early onset of non‐oxidative energy usage. This may also offer a helpful pathway for identifying dysfunction in clinical decision making so that people are appropriately diagnosed and diverted into the support pathway that is specific to the needs of the individual. The findings of the present study align with the above model, and in the subsequent sections, the potential cardiovascular, pulmonary and oxidative alterations that could underpin the observations of this study will be evaluated.

### 4.1 Cardiac and muscle oxidative limitations

The present study demonstrated that a single bout of sub‐maximal exercise led to a reduction in oxygen pulse and oxygen consumption, indicating a potential impairment in cardiac and peripheral skeletal muscle function. Other cardiovascular measures (i.e., HR and BP) at rest and HR at ventilatory thresholds were unchanged between CPET days. A systematic review and meta‐analysis reported cardiac limitations were uncommon in people living with long COVID; however, detailed cardiac testing has found reduced stroke volume augmentation that was likely attributable to preload failure (Durstenfeld et al., 2022). Invasive CPET has observed distinct endotypes of long COVID, including those that present with ventilatory limitations, decreased oxygen extraction with and without preload insufficiencies, deconditioning, and exercise pulmonary hypertension (Risbano et al., 2023). Gattoni et al. (2025) recognised that from the presenting symptoms in their cohort (*n* = 15) that there was an absence of participants presenting with autonomic dysfunction, and more purposeful recruitment in future research may enable a better representation of people living with long COVID. The findings of the present study were gathered from a larger cohort than that of Gattoni and colleagues (2025) and may be more inclusive of the multiple long COVID endotypes, which may have revealed the significant reductions in CPET parameters. Data from Gattoni et al. (2025) were also representative of a cohort where 80% met the DePaul symptom screening questionnaire definition for PEM, and it is uncertain whether PEM or PESE was observed. Conversely, the present study excluded those individuals with moderate–severe risk PEM determined by the same scale, and included participants who had mild–moderate risk PEM and/or reported PESE, defined as a worsening of symptoms that occurs after any exertion above a personal and variable tolerance threshold (Thaweethai et al., 2023). Colosio et al. observed measures within the normal range for cardiac function but did report limited exercise capacity in people with long COVID due mainly to peripheral limitations (Colosio et al., 2023). These included lower muscle oxidative capacity assessed through near‐infrared spectroscopy (NIRS) and substantial reductions of mitochondrial function biomarkers, inclusive of citrate synthase, peroxisome proliferator‐activated receptor γ coactivator 1‐α, and $J_{O_{2}}$ for mitochondrial complex II in those with long COVID compared to a control group. Appelman et al. (2024) have demonstrated a lower power output at VT1 in people living with long COVID compared with controls and observed a reduction in peripheral oxygen extraction. Using NIRS of the vastus lateralis muscle, changes of muscle deoxygenation relative to maximum indicated lesser peripheral oxygen extraction in people with long COVID. Further use of NIRS technology in 2‐day CPET may help determine whether poorer oxygen uptake or extraction worsens 24 h later and drives symptoms of PESE. NIRS also offers a far less invasive method compared with muscle biopsies that could be better tolerated by people living with long COVID.

### 4.2 Ventilatory limitations

The present study found no significant differences between breathing frequency or minute ventilation between any established thresholds, and baseline assessments of respiratory function and strength indicated normal function. However, a significant decrease in $P_{\mathrm{ETC}O_{2}}$ was observed during the second day at rest and at VT1. By contrast, lower maximal ventilation and lower maximal $P_{\mathrm{ETC}O_{2}}$ have been found in people living with long COVID compared with controls, which implied poorer ventilatory function during exercise (Appelman et al., 2024); meanwhile, dysfunctional breathing and elevated ${\dot{V}}_{E}/{\dot{V}}_{CO_{2}}$ as a measure of ventilatory inefficiency (which can be increased due to increased dead space (high $\dot{V}$/$\dot{Q}$) and/or hyperventilation) have also been reported from CPET with people living with long COVID (Durstenfeld et al., 2022). Likewise, hyperpolarised xenon magnetic resonance imaging has been used to observe regional reductions in pulmonary perfusion in those presenting with lung abnormalities following SARS‐CoV‐2 infection, including pulmonary thrombosis, fibrosis, thromboembolism and small airways disease (Wild et al., 2024). The functional consequences of these structural differences are an impaired diffusing lung capacity for carbon monoxide, which consequently impairs O_2_ diffusion at the pulmonary level (Fortini et al., 2022). Heightened dyspnoea was reported in the present study through the MRC dyspnoea scale, albeit the severity of this symptom varied between participants from only breathless during strenuous exercise to having to stop on the level to manage the symptom when walking at their own pace. Given the heterogeneity of acute COVID‐19 infections and presenting long COVID symptoms (Davis et al., 2021), it is possible that ventilatory limitations may be part of the multi‐mechanistic aetiology that perturbs a select proportion of people living with long COVID. Accordingly, more research is needed to examine the involvement of the respiratory system that may be limiting not only ${\dot{V}}_{O_{2}}$ during exercise but also functional, everyday activities.

### 4.3 Limitations

There was a lack of an appropriate control group free from any confounding comorbidities (i.e., ME/CFS, COPD, etc.) in this study. The addition of a similar comparator group with detailed descriptions of any conditions or medications that may influence the findings following any subsequent revelations regarding long COVID mechanisms is a strong recommendation. Ethnic background was not reported during this study and is a further recommendation given how some cultural groups have been disproportionately affected by the COVID‐19 pandemic (Jaljaa et al., 2022; Kirby, 2020). Future research must ensure that all groups are proportionally represented to understand any heterogeneity that may arise from the pathophysiology and lived experience. This should be addressed within the recruitment process to ensure proportional and true representation across all domains. This not only applies to ethnicity, but also age and sex, as it is typically seen within long COVID literature that middle‐aged females are the most prevalent group (Sylvester et al., 2022; Thomas et al., 2023). Furthermore, whilst the present study provides a series of cardiorespiratory limitations that may cause persistent symptoms, detailed investigations are required to understand the complexities of the long COVID pathophysiology. This includes, but is not limited to, neurological and inflammatory responses that coordinate and bring about the physiological limitations reported. This work should also seek to examine PESE, both physiologically and through perceived scales, inclusive and beyond 24 h, possibly starting up to a week after, day‐by‐day, to profile the timeliness of PESE following exertion.

### 4.4 Conclusions

This study found that people with long COVID without moderate–severe PESE, as determined by the DePaul symptom screening questionnaire, exhibit an impaired cardiorespiratory response evident as early as the first ventilatory threshold during submaximal CPET, which worsens during an additional CPET 24 h later. Strict testing protocols restricted people living with long COVID to submaximal intensities for their safety; however, significant perturbations of normal physiological processes are clear and not limited to perceptual responses, and these findings should be used to direct future research into the treatment and management strategies to improve patient outcomes. Supplementary information.

## AUTHOR CONTRIBUTIONS

Mark A. Faghy, Ruth E. Ashton, Thomas Bewick, Tom Maden‐Wilkinson, Ross Arena, Cemal Ozemek, Federico Formenti, Sundar Kumar Veluswamy, and Rachita Gururaj conceived the idea and design of the study. Callum Thomas, Nik Kudiersky, Paul Ansdell, and Emily Hume led data collection at respective test sites. Ruth E. Ashton, Calum Brown, Jack Carr, Padraig Spillane, Elisa Pastorio, Rebecca Owen, Tom Maden‐Wilkinson, Ethan McNeil‐Angopa, Tom Parkington, and Mark A. Faghy all contributed to data collection. Data analysis was performed by Callum Thomas, Nik Kudiersky, and Emily Hume. Callum Thomas and Mark A. Faghy led the write‐up of the submitted manuscript. All partners contributed to the development of the submitted manuscript. All authors have read and approved the final version of this manuscript and agree to be accountable for all aspects of the work in ensuring that questions related to the accuracy or integrity of any part of the work are appropriately investigated and resolved. All persons designated as authors qualify for authorship, and all those who qualify for authorship are listed.

## CONFLICT OF INTEREST

No potential competing interest was reported by the authors.

## Supporting information



Supporting information provides raw, anonymised demographic, physiological, and CPET participant data.

## Data Availability

Anonymised data can be located in Supporting Information
